# Exploring Saliva as a Sample for Non-Invasive Glycemic Monitoring in Diabetes: A Scoping Review

**DOI:** 10.3390/biomedicines13030713

**Published:** 2025-03-14

**Authors:** Patricia Sthefani Calixto, Fernanda Cereda Ferraz, Gabriela Carolina Dutra, Maria Julia Belotto Pelozzo, Mariana Eleni Trovão, Fabiane Gomes de Moraes Rego, Geraldo Picheth, Patrícia Maria Stuelp Campelo, Marcel Henrique Marcondes Sari

**Affiliations:** 1 Graduate Program in Pharmaceutical Sciences, Department of Clinical Analysis, Federal University of Paraná, Curitiba 80210-170, PR, Brazil; pati.sthefani@gmail.com (P.S.C.); rego@ufpr.br (F.G.d.M.R.); geraldopicheth@gmail.com (G.P.); 2 Medical Course, Pontifical Catholic University, Curitiba 80215-901, PR, Brazil; fefinha2525@gmail.com (F.C.F.); gabrielacarolinadutra@gmail.com (G.C.D.); mariajulia.bpelozzo@gmail.com (M.J.B.P.); maritro04@hotmail.com (M.E.T.); 3 School of Medicine and Life Sciences, Pontifical Catholic University, Curitiba 80215-901, PR, Brazil; pmscampelo@gmail.com

**Keywords:** diabetes mellitus, salivary glucose, glucose monitoring, plasma glucose, non-invasive tool

## Abstract

**Background/Objectives**: Diabetes mellitus is characterized by a dysregulated glucose metabolism, necessitating frequent and often invasive monitoring techniques for its effective management. Saliva, a non-invasive and readily accessible biofluid, has been proposed as a potential alternative for glycemic monitoring due to its biochemical correlation with blood glucose levels. This scoping review aims to evaluate the evidence regarding the use of salivary glucose as a biomarker to track glycemic changes in diabetic populations. **Methods**: This study adhered to the Joanna Briggs Institute guidelines and the PRISMA Extension for Scoping Reviews. A literature search was performed across the PubMed, Scopus, and Web of Science databases, supplemented by manual searches. **Results**: A total of fifty-seven studies were included, representing populations affected by type 1 diabetes (T1D), type 2 diabetes (T2D), and gestational diabetes (GD). The findings indicated consistent positive correlations between the salivary and blood glucose levels in most studies, although there were significant variations in the sensitivity, specificity, and methodological approaches. Salivary glucose showed promise as a complementary biomarker for glycemic monitoring, particularly due to its non-invasive nature. **Conclusions**: Challenges such as variability in salivary composition, the absence of standardized collection protocols, and the limited availability of portable devices were noted. This review highlights the potential of saliva as an adjunct sample for diabetes management while stressing the need for further research to bridge existing gaps.

## 1. Introduction

Diabetes mellitus (DM) is a group of metabolic disorders encompassing three main subtypes: type 1 diabetes (T1D), typically diagnosed in children and adolescents, characterized by autoimmune destruction of the beta cells, leading to absolute insulin deficiency. This category includes cases such as Latent Autoimmune Diabetes in Adults (LADA) [1,2,3]. Type 2 diabetes (T2D), the most prevalent form, is primarily driven by lifestyle and genetic factors. It is characterized by a progressive loss of adequate insulin secretion by the beta cells without autoimmune involvement, often associated with insulin resistance [1,2,3]. Gestational diabetes (GD), diagnosed in the second or third trimester of pregnancy, refers to any degree of glucose intolerance identified during gestation that was not overt diabetes before pregnancy. Moreover, other forms of diabetes, including T1D, may manifest during pregnancy [1,2].

Effective DM management requires significant lifestyle adjustments, with regular self-monitoring of capillary blood glucose being a cornerstone of disease management [4,5]. Traditionally, diabetes is diagnosed and monitored through a combination of laboratory tests, physical examinations, and evaluations of medical history [6]. According to the American Diabetes Association (ADA)’s guidelines, DM is diagnosed if a person’s fasting glucose levels are ≥126 mg/dL, their glycated hemoglobin (Hb_A1c_) levels are ≥6.5%, or their two-hour plasma glucose value after a 75 g Oral Glucose Tolerance Test (OGTT) is ≥200 mg/dL [1]. Among these methods, Hb_A1c_ is considered the gold standard, reflecting long-term glucose exposure through hemoglobin glycation [7,8,9].

Despite their proven efficacy, these conventional blood glucose monitoring techniques present notable challenges. This is primarily due to their invasive methodology, which can lead to discomfort and complications for patients [10]. Repetitive capillary blood sampling through finger-pricking can lead to significant discomfort and pain. Over time, this practice may result in complications including the development of calluses, compromised circulation, and a heightened risk of infections, especially when stringent hygiene protocols are not adhered to [11,12,13]. Additionally, Hb_A1c_ testing requires specialized equipment and costly reagents, limiting its accessibility, especially in low- and middle-income regions [8,14].

Saliva has increasingly been recognized as a viable non-invasive alternative for the monitoring of DM, particularly due to its composition being influenced by metabolic regulatory changes [15,16,17]. Accordingly, diabetes-induced changes in the autonomic regulation of the salivary glands affect the acinar and ductal cells, resulting in measurable changes in salivary composition. The secretory function of various exocrine glands, such as the salivary glands, is closely linked to perfusion, facilitating the role of saliva as a partial filtrate of the plasma and serving as a biomarker for an individual’s health status. Moreover, saliva presents significant advantages over blood-based monitoring for metabolic assessments, encompassing the ease of collection, reduced contamination risk, and the elimination of invasive sampling procedures [18,19]. This emerging utility underscores the potential of salivary analysis in monitoring DM effectively.

In patients with DM, alterations in the structural integrity of the vascular basal membrane enhance the translocation of glucose from the blood to the saliva, resulting in an elevated concentration of glucose within the salivary fluid [4,20]. Hyperglycemia, characteristic of diabetes, promotes the production of advanced glycation end products (AGEs), which crosslink collagen and extracellular matrix proteins, causing endothelial dysfunction and increased basal membrane permeability. These mechanisms explain the elevated glucose concentration in the saliva of diabetic patients. Moreover, the salivary glands play a role in blood glucose regulation, influenced by hormonal and neural mechanisms, especially in stressful situations [4,20]. Although the concentration of glucose in the saliva is roughly one-twentieth that in the blood, recent advancements in assay technologies have markedly enhanced the sensitivity and accuracy of glucose detection at these lower concentrations [18]. Furthermore, these technological improvements have facilitated the identification of related biomarkers such as proteins, enzymes, and ions, thereby contributing to a more comprehensive understanding of metabolic fluctuations in individuals with diabetes [19,21]. Lastly, reinforcing the use of saliva, several studies have shown a significant correlation between salivary glucose levels and critical blood parameters such as fasting glucose, Hb_A1c_, and other biochemical markers of metabolic control [4,15,22]. This correlation endorses the potential of saliva as a fluid that mirrors glycemic changes and offers a practical and rapid alternative for patient monitoring, along with the added benefit of being less invasive [4,15].

This scoping review analyzes primary research exploring the potential of salivary markers as a practical and non-invasive tool for monitoring glycemic changes in people with diabetes. By examining the utility of salivary biomarkers in reflecting glycemic changes, this review seeks to highlight their role in improving glucose monitoring and contributing to the development of accessible, patient-friendly methods. To the best of our knowledge, this study is the first scoping review to comprehensively compile and critically analyze the use of saliva for diabetes management, offering a unique perspective on its potential as a non-invasive monitoring sample. This study underscores the significant variations in the methodologies and populations across the included studies, reflecting the complexity and potential of using saliva for monitoring DM. Overall, more than 50 articles were evaluated in detail; to provide a focused analysis, the following sections present an overview of the features of the main selected studies (year of publication, country, diabetes type, sampling method and patient preparation, biomarkers assessed, and glucose determination method) and discuss them based on the type of diabetes (T1D, T2D, GD, and pre-diabetes). Each section highlights the key findings, correlations, and the utility of saliva as a monitoring strategy, contributing to a comprehensive understanding of its potential in diabetes management.

## 2. Results and Discussion

Despite advances in diabetes treatment, many patients still face challenges in continuous glucose monitoring, and the reliance on invasive techniques can compromise treatment adherence and impact the effectiveness of disease management [10,11,13]. In this context, this section explores the feasibility of saliva as a sample for evaluating metabolic oscillations in DM. The objective is to examine scientific evidence on the relationship between salivary glucose and blood glucose, highlighting its applicability in diabetes monitoring. Initially, the results of the search strategy adopted in the selection of the studies are presented, with a textual description and tables summarizing the most relevant information from each article. Subsequently, research on the use of saliva for glycemic monitoring is analyzed and organized in a stratified manner according to the different types of diabetes to facilitate an understanding of the findings and their clinical implications.

### 2.1. General Findings and Descriptive Statistics

**Figure 1:**
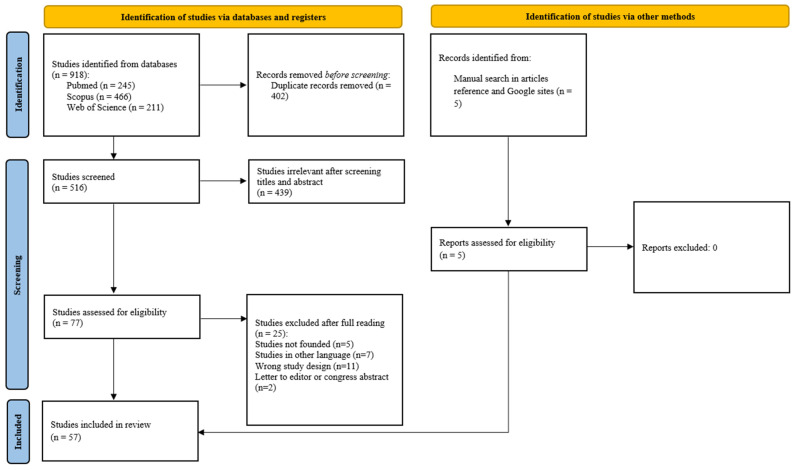
Flowchart of the study selection process.

**Figure 2:**
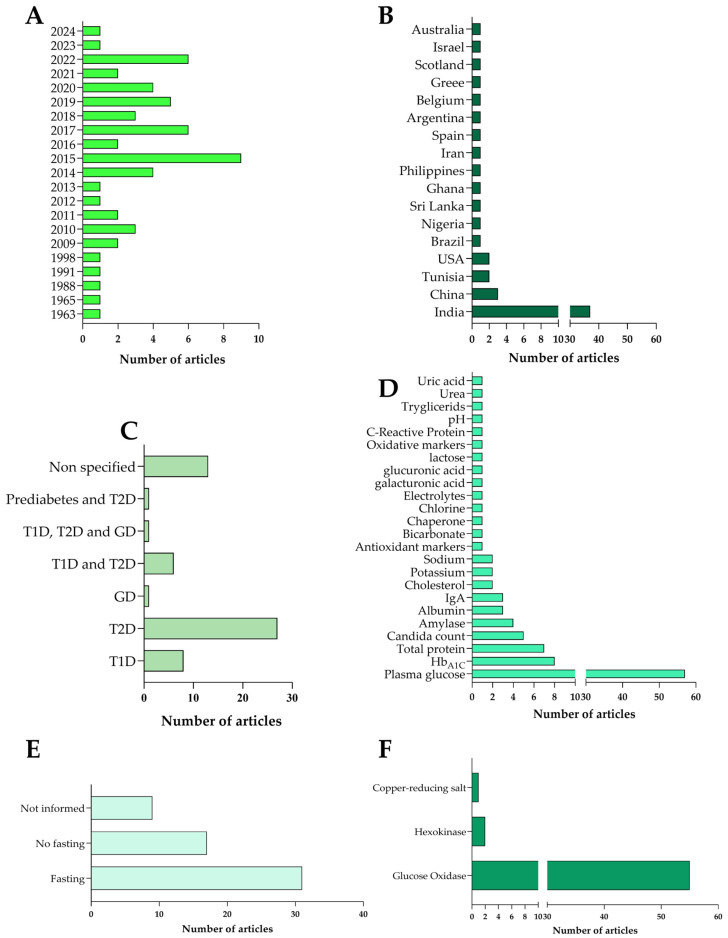
A detailed descriptive statistical analysis of the selected studies was conducted, focusing on the year of publication (**A**), country (**B**), population (**C**), biomarkers (**D**), patient preparation (**E**), and glucose assessment methodology (**F**).

The search strategy yielded a total of 918 articles, from which 516 studies were subjected to screening after the elimination of duplicates (Figure 1). Following this review, 402 records were excluded due to a lack of relevance, resulting in the selection of 77 studies for full-text evaluation. Of these, 25 studies were excluded based on predefined eligibility criteria. Additionally, 5 studies identified through manual searching were incorporated, leading to 57 studies being included in the final analysis. Figure 2 depicts a summary of the main features of the selected studies to improve their visualization and comprehension.

Approximately 61% originated from India (*n* = 37/57), followed by contributions from China (*n* = 3), Tunisia (*n* = 2), the USA (*n* = 2), and one study each from Brazil, Nigeria, Sri Lanka, Ghana, the Philippines, Iran, Spain, Argentina, Belgium, Greece, Scotland, Israel, and Australia (Figure 2A). These studies, conducted between 1963 and 2024 (Figure 2B), primarily investigated case–control clinical trials exploring the relationship between salivary glucose levels and glycemic parameters in people with and without diabetes.

Table 1 summarizes the key characteristics of the studies included. The sample sizes varied between 20 and 600 participants, including diabetic and non-diabetic individuals, with a balanced distribution of genders. Most studies concentrated on patients with T2D (*n* = 27/57), followed by patients with T1D (*n* = 8/57), and one study focused on patients with GD. Six studies incorporated T1D and T2D patients, while another examined a sample that included T1D, T2D, and GD patients. Additionally, one study focused on individuals with T2D and pre-diabetes. Thirteen studies did not specify the type of diabetes represented (Figure 2C).

**Figure 3:**
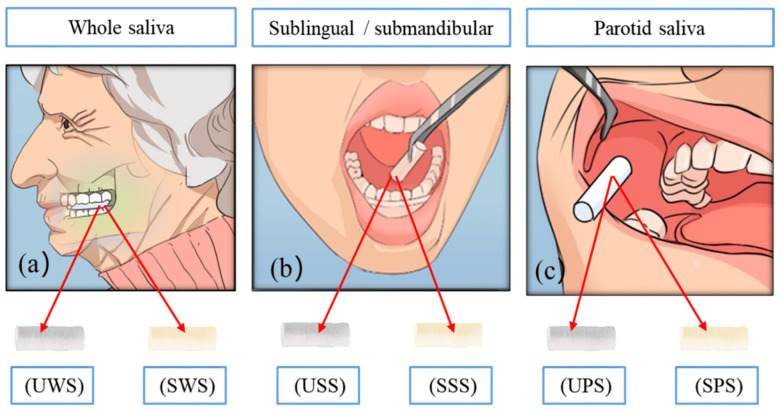
Methods for collecting saliva samples. (**a**) Whole saliva collection; (**b**) collection of sublingual/submandibular salivary secretions; and (**c**) parotid saliva collection. Abbreviations: UWS: unstimulated whole saliva; SWS: stimulated whole saliva; USS: unstimulated sublingual/submandibular saliva; SSS: stimulated sublingual/submandibular saliva; UPS: unstimulated parotid saliva; SPS: stimulated parotid saliva. Data available in [4].

The saliva collection predominantly involved non-stimulated (spontaneous) samples (*n* = 49/57). Other studies utilized both spontaneous and stimulated samples, with citric acid (*n* = 5/57), mastication (*n* = 1/57), and paraffin tablets (*n* = 1/57) as the stimulants. One study exclusively employed lemon juice for stimulation. The methods for saliva collection included spitting (*n* = 41/57), drooling (*n* = 7/57), aspiration (*n* = 5/57), and absorption (*n* = 3/57). One study did not report its method of saliva collection. Considering such aspects regarding the saliva sampling, a robust comparison among the studies is impaired. Saliva is a complex fluid composed of secretions from different glands (parotid, submandibular, and sublingual) (Figure 3). Its composition is dynamic and influenced by factors such as the collection technique and the individual’s health. Furthermore, saliva can be total or specific to a single gland, and its glucose content varies depending on its origin. Studies indicate that parotid salivary glucose is higher than that in the total and sublingual saliva, but research on specific glands is still limited [4]. Sterile plastic containers (*n* = 54/57) were the most common collection vessels, followed by sterile cotton swabs (*n* = 3/57). Five studies added fluoride to the samples to inhibit glucose oxidation. The pre-collection guidelines often included instructions to rinse one’s mouth, avoid dental procedures, refrain from smoking or chewing gum, and maintain fasting conditions. Fasting was required in 31 studies (*n* = 31/57), 17 did not specify fasting status, and 9 provided no information on this aspect (Supplementary Materials) (Figure 2E).

Table 2 highlights the key characteristics of the studies included and provides a detailed analysis of the participants’ serum and salivary fasting glucose levels (if available). These values are categorized according to each research study and further organized by the type of diabetes, offering a comprehensive overview of the data.

For glucose determination, the glucose oxidase method was predominantly employed (*n* = 55/57), with hexokinase (*n* = 2/57) and a generic copper-reducing salt method (*n* = 1/57) used less frequently (Figure 2F). This comprehensive analysis highlights the methodological variations and points to the potential challenges in using salivary glucose as a monitoring tool for diabetes management (Supplementary Materials).

### 2.2. Salivary Glucose Monitoring in T1D: Evidence and Perspectives

T1D is an autoimmune disease characterized by the destruction of the insulin-producing beta cells in the pancreas by the immune system, leading to absolute insulin deficiency and chronic hyperglycemia. T1D predominantly affects children and young adults, with an estimated 9 million people globally living with this disease. While it is less common than T2D, T1D poses significant public health challenges due to its lifelong need for insulin therapy and meticulous glucose monitoring. Its economic burden is considerable, encompassing direct costs such as insulin, glucose monitoring supplies, and hospitalizations, as well as indirect costs related to lost productivity and complications from inadequate glycemic control [75,76,77].

The traditional management of T1D relies heavily on blood glucose monitoring, which involves frequent finger pricks. Although this method is effective, it is often associated with pain, discomfort, and reduced adherence, particularly among children and adolescents. These challenges have spurred interest in alternative monitoring methods, with saliva-based glucose determination emerging as a promising, non-invasive, painless, and accessible option. Saliva can improve patient compliance and provide a practical alternative for continuous glucose monitoring [13,55,78,79].

Somes studies have explored the potential of saliva as a diagnostic tool in T1D populations, particularly in individuals under 20 years of age [27,33,38,42]. Arora et al. [41] and Ganesan et al. [70] focused on young adults, while only Cheprasova et al. [71] exclusively included adults. Interestingly, T1D exhibits a male gender bias, with higher incidence rates in men, especially post-puberty [80,81]. This discrepancy has been linked to the transient protective role of the female gonadal hormones during puberty [82,83].

Biochemical changes in the saliva of T1D patients have shown significant promise for monitoring purposes. Elevated salivary glucose levels strongly correlate with hyperglycemia observed in the serum [27], and similar trends have been noted for other biomarkers, such as total protein, cholesterol, and triglycerides [71]. Positive correlations between fasting serum and salivary glucose levels have been consistently reported, with correlation coefficients (r) ranging from 0.65 to 0.99 and statistically significant values (*p* < 0.05) [33,38].

Some studies, however, have reported inconsistencies. For example, Ghena et al. [34] found no significant correlation between serum and salivary glucose in diabetic patients but identified a strong correlation in the controls. These findings suggest that factors such as ongoing treatment or variability in the study designs may influence salivary glucose levels. Additionally, smaller sample sizes and methodological differences may contribute to discrepancies in the findings across studies.

Other biomarkers, such as salivary albumin and total protein, have shown significant elevations in T1D patients [38,42]. Elevated levels of inflammatory markers, including CRP and IL-6, advanced glycation end products (AGEs), and altered electrolytes levels (sodium and potassium) [71] have been noted. These comprehensive biomarker profiles could enhance our understanding of a patient’s glycemic status and overall health.

While some studies have reported weakness [42], most findings underscore the feasibility of saliva as a useful sample for glycemic monitoring in T1D patients. By addressing the limitations of blood-based tests, saliva-based methods offer a novel, patient-friendly approach that could revolutionize diabetes management, particularly for populations requiring frequent monitoring. Further research is essential to validate these findings and establish standardized protocols for their clinical application. This innovative approach could improve the quality of life for T1D patients while maintaining effective glycemic control.

Therefore, this body of evidence demonstrates the potential of saliva as a non-invasive and practical tool for monitoring glycemic levels in T1D populations. The studies included in this review reveal significant correlations between salivary and serum glucose levels and other biomarkers. However, limitations such as variability in the methodologies, small sample sizes, and a lack of standardized protocols restrict definitive conclusions. Future research should prioritize larger, well-controlled cohorts to validate these findings, investigate the influence of the treatment regimens, and establish standardized protocols for saliva collection and analysis. Addressing these gaps could position saliva as a transformative tool for T1D management, enhancing patient comfort and adherence while maintaining effective glycemic control.

### 2.3. Type 2 Diabetes (T2D) Monitoring Through Salivary Biomarkers

Unlike T1D, T2D is primarily characterized by insulin resistance and persistent hyperglycemia [59]. It affects millions of individuals worldwide and has reached epidemic proportions [81]. The prevalence of T2D varies significantly across regions, influenced by factors such as lifestyle, diet, and genetic predisposition [84,85,86,87,88]. According to the World Health Organization, over 400 million people are currently living with diabetes, with T2D accounting for approximately 90% of all cases [84,89]. Both impose a significant economic burden on healthcare systems, driven by direct costs—including hospitalizations, medications, and outpatient care—and indirect costs related to lost productivity and disability [90].

Monitoring glucose levels is a cornerstone of effective diabetes management. Traditionally, this has been achieved through blood sampling, which, despite its effectiveness, has notable limitations. Frequent finger pricks can cause discomfort, increase the risk of infection, and discourage consistent monitoring, especially among patients requiring regular glucose checks. These challenges have led to growing interest in non-invasive methods, such as salivary glucose monitoring, as an alternative to blood-based approaches [10].

Two primary methods for saliva collection are unstimulated and stimulated collection. Unstimulated collection allows the saliva to accumulate naturally in the oral cavity without external stimulation or manipulation. In contrast, stimulated collection is employed when a larger volume of saliva is needed for analysis. This method encourages salivary flow through various stimuli, such as chewing paraffin wax or gum or using gustatory substances like citric acid. These stimuli significantly enhance saliva production, making it easier to collect an adequate volume for analytical purposes [10].

Saliva stimulation alters its composition, leading to differences between unstimulated and stimulated saliva. Research indicates that unstimulated saliva may be more suitable for monitoring applications, as stimulated saliva can cause inaccuracies in measuring its constituents due to the increased dilution caused by stimulation [43,91]. Additionally, using external substances to stimulate saliva production modulates the fluid’s pH and favors the aqueous phase’s secretion, resulting in lower concentrations of target proteins [92].

In our analysis of studies investigating the use of saliva for monitoring T2D, only two employed stimulated saliva collection methods. Carramolino-Cuéllar et al. [54] used a paraffin tablet for stimulation but did not report significant differences between the stimulated and unstimulated saliva collection methods. Sashikumar et al. [28] used citric acid as a stimulant and observed a positive correlation between stimulated and unstimulated saliva and random, non-fasting plasma glucose levels. Notably, the correlation was slightly higher for stimulated saliva (r = 0.635 for unstimulated saliva versus r = 0.686 for stimulated saliva), suggesting a potential enhancement in the reliability of measurement with stimulation.

Saliva offers unique advantages due to its non-invasive nature and ease of collection [93]. In patients with T2D, salivary glucose levels are frequently elevated and correlate with hyperglycemia [94]. Several studies have investigated the correlation between salivary glucose and blood glucose in individuals with T2D and healthy controls. Notably, Satish et al. [22], Choudhry et al. [72], Ghafouri et al. [56], Harish et al. [58], Gupta et al. [40], Abikshyeet et al. [35], Mrag et al. [64], Balan et al. [36], Mishra et al. [60], Dhanya et al. [49], Puttaswamy et al. [50], and Kumar et al. [37] reported a positive correlation in both groups. Conversely, Dharmakeerthi et al. [67], Shaik et al. [53], Abd-Elraheem et al. [52], Wang et al. [51], Hegde et al. [30], and Mussavira et al. [47] found a positive correlation only in the T2D group. Additionally, Bhattacharyya et al. [57], Tiongco et al. [15], Carramolino-Cuéllar et al. [54], Sashikumar et al. [28], and Ravindran et al. [43] observed a positive correlation when the T2D and control groups were analyzed together. The reported correlation coefficient (r) values ranged from 0.201 to 0.929 in the T2D group and from 0.185 to 0.9 in the control group, with statistical significance (*p* < 0.05). These findings suggest a consistent association between salivary glucose and blood glucose levels, particularly in individuals with T2D, reinforcing saliva’s potential as a non-invasive glycemic monitoring sample.

Among the analyzed studies, in Shaik et al. [53], Satish et al. [22], Choudhry et al. [72], Harish et al. [58], Abikshyeet et al. [35], Dharmakeerthi et al. [67], Ghafouri et al. [56], Wang et al. [51], Hegde et al. [30], Mrag et al. [64], Ravindran et al. [43], Mussavira et al. [47], Dhanya et al. [49], Carramolino-Cuéllar et al. [54], Tiongco et al. [15], and Puttaswamy et al. [50], the saliva samples were consistently collected in a fasting state. In the T2D group, the serum glucose levels ranged from 96.62 to 230.07 mg/dL, while in the control group, these values ranged from 74.75 to 101.61 mg/dL. The salivary glucose levels for the T2D group varied from 0.4 to 16.3 mg/dL, whereas these levels in the control group ranged from 0.2 to 7.41 mg/dL.

Kumar et al. [37], Balan et al. [36], and Mishra et al. [60] applied non-fasting samples. In the T2D group, the serum glucose levels ranged from 137 to 355.83 mg/dL, while in the control group, they ranged from 93 to 112.6 mg/dL. The salivary glucose levels in the T2D group varied from 3 to 13.35 mg/dL, whereas in the control group, they ranged from 1.18 to 9.46 mg/dL.

A noteworthy observation is that some studies subdivided the T2D group into controlled and uncontrolled diabetes patients [36,37,47,58,60]. All studies reported higher glucose concentrations in both the saliva and blood among T2D patients, along with a positive correlation between these two variables. Interestingly, Harish et al. [58], Kumar et al. [37], and Mussavira et al. [47] found a stronger correlation in the uncontrolled diabetes group (r = 0.917–0.929, *p* < 0.001) compared to that in the controlled group (r = 0.748–0.9, *p* < 0.001). In contrast, Balan et al. [36] and Mishra et al. [60] observed a more robust correlation in the controlled diabetes group (r = 0.43–0.896) than that in the uncontrolled group (r = 0.3–0.861).

These findings suggest that glycemic control status may influence the strength of the relationship between salivary and blood glucose concentrations. Shettigar et al. [74] reported a weak correlation (r = 0.4, Spearman’s) between blood and salivary glucose levels in diabetic patients. Based on these results, the authors concluded that saliva is better suited as a monitoring tool rather than a diagnostic tool, particularly for patients with elevated glycemic levels. Similarly, Shaik et al. [53] emphasized the utility of saliva for regular follow-ups in individuals already diagnosed with T2D rather than for diagnosing those with an unknown glycemic status. This perspective is further supported by the findings from Wang et al. [51], which highlighted the potential of salivary glucose as a non-invasive indicator for monitoring T2D, contributing to the effective management of diabetic patients.

In contrast to previous findings, Vasconcelos et al. [31], Egboh et al. [69], and Indira et al. [45] did not identify a positive correlation between salivary glucose and blood glucose levels. The conclusions of these studies diverge: Indira et al. [45] emphasize the need for further research involving larger populations and diverse regions to evaluate the potential of salivary glucose as a monitoring sample for diabetes. Conversely, Vasconcelos et al. [31] conclude that salivary glucose levels are not influenced by blood glucose and therefore are unsuitable for monitoring glycemic control in diabetic patients. Additionally, the concentration of salivary glucose shows significant variability across studies. This disparity may be attributed to differences in the methods of collection, processing, and analysis of the salivary samples, emphasizing the need for methodological standardization in future research [57].

Some studies that observed a positive correlation between salivary and blood glucose levels calculated the regression coefficient, which quantifies the magnitude of the change in blood glucose for each unit of variation in salivary glucose. This coefficient enables the prediction of blood glucose levels based on specific salivary glucose values through regression equations [49]. The Supplementary Materials present the expected increase in blood glucose for every 1 mg/dL increment in salivary glucose, as reported in the analyzed studies. These values range from 1.63 mg/dL to 15.7 mg/dL, demonstrating the variability across studies and underscoring the potential of this approach as a predictive tool for estimating blood glucose levels.

Several studies have investigated the sensitivity and specificity of salivary glucose in detecting diabetes, establishing different cutoff values. Kumar et al. [35] reported that diabetes could be identified with a salivary glucose concentration above 6.8 mg/dL, yielding a sensitivity of 83.33% and a specificity of 100%. Conversely, Mrag et al. [64] suggested a lower cutoff value of 4.50 mg/dL, with a sensitivity of 78% and a specificity of 80%. In contrast, Tiongco et al. [15] identified a higher cutoff value of 13.22 mg/dL, associated with a sensitivity of 76% and a specificity of 90%. These findings highlight the variability in the salivary glucose cutoff values, differences which may have been influenced by the study methodologies and population characteristics.

Hb_A1c_ has been widely used to diagnose DM and monitor glycemic control in recent months. It is also relevant for validating the use of salivary glucose as a monitoring marker. Satish et al. [22] and Choudhry et al. [72] identified a positive correlation between salivary glucose and Hb_A1c_ levels in diabetic and control groups. However, Kumar et al. [37], Dharmakeerthi et al. [67], Abikshyeet et al. [35], and Gupta et al. [40] demonstrated this correlation only in the T2D group. In contrast, Harish et al. [58] reported a significant correlation exclusively in the uncontrolled diabetic group. In the diabetic groups, their Hb_A1c_ levels ranged from 5.96% to 9.99%, with correlation coefficients (r) between 0.277 and 0.906 (*p* < 0.05), reinforcing the relationship between salivary glucose and Hb_A1c_ as complementary markers for glycemic status.

Additionally, saliva reflects broader biochemical changes associated with diabetes, including alterations in amylase activity, increased inflammatory markers such as CRP and IL-6 levels, changes in IgA, and electrolyte content [95]. These biomarkers provide a holistic view of a patient’s glycemic status and overall health, reinforcing the potential of saliva as a comprehensive monitoring tool. Indira et al. [45] reported significantly lower mean salivary amylase levels in diabetic patients (10,766 U/L) compared to those in the control group (15,496 U/L), potentially due to hormonal and metabolic alterations associated with diabetes. Additionally, a significant positive correlation was identified between salivary amylase and total protein levels, explained by the fact that amylase is a component of total protein, meaning that changes in its concentration directly impact total protein levels.

Conversely, Abd-Elraheem et al. [52] and Mrag et al. [64] reported significantly higher amylase levels in diabetic patients (2164.3 U/L and 143,461.5 U/L, respectively) compared to those in the control group (885 U/L and 1638 U/L, respectively). These discrepancies highlight the need to consider methodological variations and differences in study populations when interpreting salivary amylase data. Three studies investigated the total protein levels in the saliva of diabetic patients, demonstrating higher concentrations in the DM group compared to those in the control group. The values found were 91.80 mg/dL, 0.48 g/L, and 375.12 mg/dL in the DM group, while in the control group, they were 103.10 mg/dL, 0.195 g/L, and 202.23 mg/dL, as reported by Indira et al. [45], Mrag et al. [64], and Mussavira et al. [47], respectively. Furthermore, Indira et al. [45] identified a significant correlation between salivary glucose and total protein levels (r = −0.5181, *p* < 0.05), indicating that changes in glucose levels may be associated with changes in the protein levels in saliva.

This innovative approach to monitoring could significantly enhance patient compliance and comfort, addressing many limitations associated with traditional blood-based testing. By offering a painless, practical, and accessible alternative, salivary glucose monitoring has the potential to revolutionize T2D care and empower patients to manage their condition more effectively. However, further research is crucial to validate these findings and establish standardized protocols for clinical implementation, ensuring that saliva-based monitoring becomes an integral component of comprehensive diabetes management strategies.

### 2.4. Saliva-Based Monitoring in GD

GD is often indicative of underlying b-cell dysfunction [96], which confers a marked increased risk for the later development of glucose intolerance and diabetes in the mother after delivery [97,98]. GD affects approximately 7–10% of pregnancies globally, with its prevalence varying based on ethnicity, lifestyle, and access to healthcare [99,100]. This condition poses significant risks to both the mother and child, including preeclampsia, cesarean delivery, macrosomia, and neonatal hypoglycemia. Moreover, it increases the likelihood of T2D in mothers and the risk of obesity and diabetes in their offspring, contributing to a significant economic burden on healthcare systems and families [101,102].

The OGTT is typically used to diagnose GD between 24 and 28 weeks of pregnancy. While practical, this test presents challenges such as fasting requirements, side effects from the glucose drink, and multiple blood draws. These issues highlight the need for non-invasive and patient-friendly alternatives [75,103,104]. Saliva-based glucose monitoring emerges as a promising solution, offering a painless and convenient method that could enhance patient adherence and improve prenatal care experiences [68].

In GD, as in other forms of diabetes, salivary glucose levels often mirror the hyperglycemia observed in the blood, providing a viable biomarker for monitoring [68]. Elevated salivary glucose levels, alongside changes in amylase activity, inflammatory markers such as CRP and IL-6, and AGEs and electrolyte imbalances, create a comprehensive biochemical profile of a patient’s glycemic status. These characteristics underscore saliva’s potential as a holistic and innovative monitoring tool for GD [105,106].

A pivotal study [68] specifically focused on this type of diabetes examined 99 patients with GD and 100 healthy pregnant women. Their salivary and blood glucose levels were assessed under fasting and postprandial conditions, using both stimulated (with citric acid) and unstimulated saliva. The findings revealed significantly higher salivary glucose levels in the GD patients across all conditions compared to those in the controls (*p* = 0.001). In the GD patients, their fasting salivary glucose levels ranged from 5.37 to 6.02 mg/dL, while their postprandial levels reached up to 9.48 mg/dL. A correlation analysis demonstrated moderate positive relationships between the salivary and blood glucose levels, particularly in postprandial conditions, with coefficients of r = 0.409 (stimulated) and r = 0.414 (unstimulated).

This study also evaluated the diagnostic performance of salivary glucose, identifying cutoff values for predicting GD. Unstimulated fasting salivary glucose had a sensitivity of 58% and a specificity of 72%, with a 5.1 mg/dL cutoff. In comparison, postprandial salivary glucose showed a higher sensitivity (82%) and specificity (87–88%), with 8.8–9.3 mg/dL cutoffs. These results suggest that salivary glucose levels provide a reliable indicator for GDM diagnosis and monitoring, offering a less invasive alternative to traditional methods.

Additionally, the ability to analyze salivary glucose and correlate it with other laboratory markers commonly evaluated during pregnancy—such as lipid profiles, inflammatory markers, and hormonal levels [71]—could significantly enhance our understanding of maternal health. This integrative approach would not only address gestational diabetes but also provide valuable insights into the broader metabolic and physiological changes during pregnancy, potentially improving maternal and fetal outcomes.

Despite these promising findings, some limitations must be acknowledged. The moderate sensitivity and specificity in fasting conditions indicate that salivary glucose may not replace blood-based diagnostics entirely but could be a supplementary tool, mainly for monitoring purposes. Additionally, variability in salivary glucose levels due to external factors, such as dietary habits and sample collection methods, highlights the need for standardized protocols. More significant, multi-center studies are required to validate these findings and refine the diagnostic criteria, ensuring that saliva-based methods become integral to GD management. By addressing these gaps, saliva-based monitoring could revolutionize GD care, offering a patient-friendly, cost-effective alternative to blood-based tests. This approach not only alleviates the physical discomfort associated with the traditional methods but could also potentially improve the prenatal outcomes for mothers and their babies, fostering a new era of personalized and accessible diabetes care.

### 2.5. Salivary Glucose Monitoring in Mixed Diabetes Populations: Challenges and Insights

The composition of saliva among individuals with T1D, T2D, and GD varies significantly, reflecting the unique pathophysiological mechanisms and metabolic changes associated with each condition [25,107,108]. In T1D, acute and severe hyperglycemia often leads to markedly elevated salivary glucose levels [109]. This acute metabolic dysregulation is accompanied by increased inflammatory markers, such as IL-6 and tumor necrosis factor-alpha (TNF-α), which can also be detected in the saliva [110]. Additionally, T1D patients may experience episodes of dehydration and xerostomia (dry mouth) due to osmotic diuresis, further affecting their salivary composition [111]. As a result, these acute and immune-driven alterations are likely to produce more dynamic and variable salivary profiles than those in other types of diabetes.

In T2D, persistent insulin resistance and progressive dysfunction in the beta cells lead to sustained hyperglycemia, frequently accompanied by low-grade systemic inflammation [52,112]. This chronic condition results in consistently elevated salivary glucose levels. Furthermore, the saliva from individuals with T2D may show heightened levels of AGEs and lipids, indicating the long-term metabolic stress typical of this disease [113,114]. Over time, the salivary gland function in T2D may gradually be impaired due to ongoing metabolic disturbances, which can result in altered salivary flow rates and electrolyte imbalances [52,115]. These changes tend to be stable rather than presenting as acute fluctuations, making the salivary profile of T2D patients more consistent than that of individuals with T1D [116].

The salivary glucose levels in individuals with GD are often elevated, though they can be inconsistent due to the dynamic metabolic environment of pregnancy [117]. Moreover, GD saliva may reflect the systemic inflammation and oxidative stress characteristic of this period, evidenced by higher levels of CRP and altered enzymatic activity [118,119]. Unlike T1D and T2D, the temporary nature of GD leads to a distinct salivary profile influenced by gestational hormones, setting it apart from other types of diabetes. These differences highlight the necessity of stratifying study populations by diabetes type in the research, as its unique pathophysiological and hormonal influences on salivary biomarkers may affect the interpretation and relevance of the findings [120].

Several studies have explored the potential of salivary glucose as a monitoring tool in populations with diabetes without specifying the type of diabetes among the participants [12,23,24,25,26,30,39,46,48,61,62,66,73]. On the other hand, some studies have subdivided patients into controlled and uncontrolled diabetics [32,63] or between T1D and T2D [44,65]. Fares et al. [59] differentiated between diabetic and pre-diabetic patients. While these studies provide valuable preliminary insights, the lack of differentiation between diabetes subtypes presents notable challenges in interpreting their findings. These types differ significantly in their pathophysiology, treatment regimens, and metabolic profiles, factors that could influence salivary glucose levels and other biochemical markers. The absence of stratification by diabetes type limits the ability to identify nuances specific to T1D or T2D, such as the influence of residual insulin production in T2D or the autoimmune processes underlying T1D.

Additionally, mixed-population studies often report variations in salivary biomarkers such as total protein, albumin, and amylase, which are influenced by systemic inflammation, oxidative stress, and metabolic dysregulation [108,121,122]. These factors may differ substantially between T1D and T2D patients due to variations in the disease duration, comorbidities, and treatment strategies. Consequently, interpreting these findings without subtype differentiation risks conflating the unique characteristics of each diabetes type, potentially leading to overgeneralization.

The method used can also influence the salivary glucose levels measured. The composition of the saliva is influenced by factors such as general health and the collection method, which can be stimulated or unstimulated [123,124]. Fares et al. [59], Patel et al. [39], Hegde et al. [63], Panchbhai et al. [32], Gupta et al. [44], Ephraim et al. [61], Campbell et al. [24], Kadashetti et al. [46], Darwazeh et al. [26], Agrawal et al. [12], Pandey et al. [73], Kumar et al. [65], Smrit et al. [48], Ragunathan et al. [62], and Gupta et al. [66] opted for unstimulated saliva. However, Englander et al. [23] used saliva stimulated with lemon juice, while Ben-Aryeh et al. [25] used citric acid, observing that stimulation decreased the protein concentration in the parotid saliva in both groups (diabetics and controls), although the reduction was more pronounced in the controls. This difference may be associated with neuropathy or an altered response to cholinergic stimulation in diabetics [108,125,126].

One of the main challenges in using salivary glucose as a monitoring method for diabetic patients is the inherent instability of saliva and the influence of various external factors on salivary glucose measurements. These factors include diet, fasting status, oral hygiene, water rinsing, and whether the saliva is stimulated or unstimulated, among others [17]. The only way to mitigate these impacts is by standardizing the collection procedure to minimize the interference of these variables [17].

Cui et al. [4], comparing six collection methods, analyzed different types of saliva—whole, parotid, and sublingual/submandibular—under both stimulated and unstimulated conditions. Their results indicated that stimulated saliva, compared to unstimulated saliva, showed reduced glucose levels and increased salivary flow. Among the methods evaluated, unstimulated parotid saliva (UPS) demonstrated the highest correlation with blood glucose levels (r = 0.9153). Therefore, UPS collected before breakfast emerges as a promising non-invasive complementary method for glucose monitoring in patients with diabetes mellitus.

In another study, Cui et al. [17] demonstrated that the stimulation conditions and freezing/thawing cycles influence salivary glucose levels. However, storing samples at –20 °C for up to 35 days with a maximum of three freezing/thawing cycles did not cause significant changes in salivary glucose levels. This finding facilitates sample transport and analysis, enhancing efficiency and reducing costs. Additionally, this study highlighted that salivary glucose levels are influenced by circadian rhythms, making the timing of collection crucial. To minimize fluctuations, collecting samples on an empty stomach and at the same time of day is recommended.

Extrinsic factors such as DM symptoms and predisposing conditions also affect salivary glucose production but do not consistently recur in the same patient. Therefore, rigorous inclusion criteria are essential to ensure the uniformity and validity of studies validating salivary glucose as a DM biomarker.

**Figure 4:**
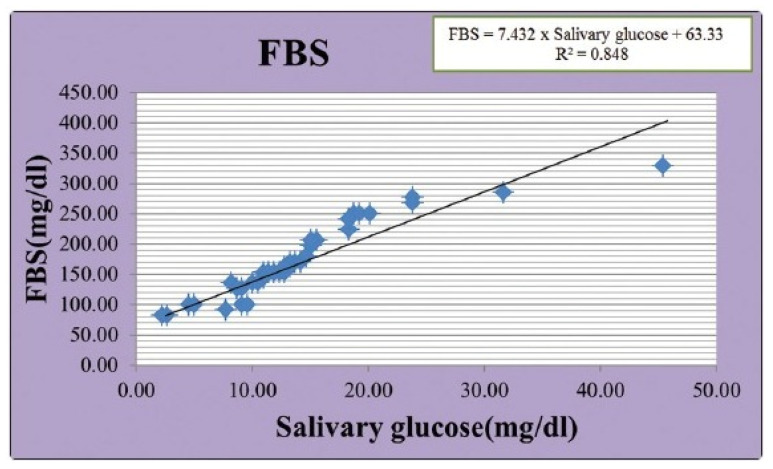
Correlation between fasting blood (FBS) glucose and salivary blood glucose. Data available in [39].

Several studies demonstrated a positive correlation between salivary and blood glucose levels, highlighting saliva’s potential as a non-invasive biomarker for glycemic monitoring [12,39,46,48,55,59,62,63,65]. Figure 4 shows the results of the correlation observed by Patel et al. [39]. In diabetic patients, the fasting serum glucose levels ranged from 111.31 to 283.23 mg/dL, compared to 78.39 to 92.11 mg/dL in the control groups. Correspondingly, the fasting salivary glucose levels in diabetic patients ranged from 4.86 to 59.32 mg/dL, while the controls exhibited levels between 0.78 and 23.40 mg/dL. For non-fasting samples, the serum glucose levels ranged from 150.07 to 365.60 mg/dL in the diabetes group and 96.69 to 131.86 mg/dL in the controls. The non-fasting salivary glucose levels in diabetic individuals varied from 1.21 to 13.6 mg/dL, while those in the controls ranged from 0.45 to 6.36 mg/dL. Correlation analyses further supported these findings, with the coefficients in diabetic groups ranging from r = 0.36 to r = 0.981 (*p* < 0.001–0.01) and in control groups ranging from r = 0.4 to r = 0.937 (*p* < 0.001–0.01).

Darwazeh et al. [26] and Pandey et al. [73] identified positive correlations between the salivary and serum glucose levels exclusively within diabetic patients, with correlation coefficients of r = 0.33 (*p* < 0.05) and ρ = 0.25, respectively. However, no significant correlations were observed in the control groups, with values of r = 0.21 (*p* > 0.05) and ρ = 0.08. These findings suggest that the salivary glucose levels may reflect glycemic changes in individuals with diabetes more closely, while such associations appear weaker or absent in non-diabetic populations. Furthermore, Ephraim et al. [61] evaluated the diabetic and control groups collectively, demonstrating a strong positive correlation between salivary and serum glucose levels (r = 0.890; *p* < 0.0001) and between salivary glucose and total capillary glucose (r = 0.870; *p* < 0.0001). Furthermore, Gupta et al. [66] focused on diabetic patients, identifying significant correlations between salivary glucose and fasting blood glucose (r = 0.651; *p* = 0.000) and between salivary glucose and postprandial blood glucose (r = 0.299; *p* = 0.046). These findings underscore the potential of saliva as a reflective biomarker of systemic glucose levels, particularly in diabetic populations.

Contrary to studies reporting positive correlations between salivary and serum glucose, Gupta et al. [44] concluded that saliva is unsuitable for monitoring blood glucose in diabetic patients. No significant correlation between the serum and salivary glucose levels was found in the diabetic group (r = 0.030; *p* = 0.699). Interestingly, a modest correlation was observed in the control group (r = 0.322; *p* = 0.049), further emphasizing the variability in the relationship between salivary and blood glucose. These findings underline the need for further investigation to understand the factors contributing to such discrepancies better, such as variations in the sample preparation, patient characteristics, and glucose determination methodologies.

Similarly, Englander et al. [23] reported higher salivary glucose concentrations in diabetic patients; however, these levels were comparable to those in the healthy group, casting doubt on the feasibility of saliva as a reliable substitute for blood in glucose monitoring. Moreover, Panchbhai et al. [32], Jurysta et al. [29], Ben-Aryeh et al. [25], and Campbell et al. [24] also failed to identify significant correlations between serum and salivary glucose levels. Collectively, these findings suggest that saliva, in its current scope of use, lacks the consistency and precision required for effective monitoring or diagnostic purposes in diabetes management. Despite these limitations, the authors of these studies consistently emphasize the importance of continued research to refine the methodologies and explore saliva’s potential as a biomarker in diabetic populations further.

Some studies have assessed the sensitivity and specificity of salivary glucose in predicting diabetes mellitus. Smriti et al. [48] plotted ROC curves, revealing an area under the curve (AUC) of 0.998, indicating an excellent discrimination capacity for identifying individuals with diabetes mellitus. The optimal cutoff point for salivary glucose was 7.05 mg/dL, achieving a sensitivity of 99.1% and a specificity of 93.7%. Similarly, Ephraim et al. [58] identified a cutoff point of 9.01 mg/dL for salivary glucose, with a sensitivity of 80.0% and a specificity of 95.0%. This study also reported cutoff points of 122.5 mg/dL for serum glucose, with the sensitivity and specificity reaching 99.0% and 100.0%, respectively, and 124.32 mg/dL for total fasting capillary glucose, achieving 100.0% sensitivity and specificity. While these findings highlight the relationship between salivary glucose levels and serum or capillary glucose levels, Ephraim et al. [61] concluded that salivary glucose lacks the diagnostic precision of blood glucose. Nonetheless, this study emphasizes the potential of salivary glucose as a non-invasive tool for monitoring glycemic control in diabetes mellitus, provided that the estimation methods and analytical techniques are further refined.

While logistically simpler, the inclusion of mixed populations has implications for clinical applications. For instance, the cutoff values for salivary glucose or other biomarkers may vary between T1D and T2D populations given their distinct metabolic and pathophysiological profiles. A one-size-fits-all approach to salivary diagnostics could result in suboptimal sensitivity and specificity, underscoring the need for stratified analyses. Future research should prioritize separating populations by diabetes type to understand how salivary biomarkers behave under differing physiological conditions better.

While the current body of evidence highlights the promise of salivary glucose as a monitoring tool, addressing the heterogeneity in diabetes populations is crucial for refining its clinical utility. Stratified studies could improve the diagnostic accuracy and provide deeper insights into the biochemical dynamics of T1D and T2D, ultimately advancing the field of non-invasive diabetes management.

### 2.6. Expert Opinion: Research Limitations and Future Prospects

The current body of research highlights the promising potential of salivary glucose as a non-invasive biomarker for glycemic monitoring in DM management. However, significant study limitations must be addressed before these findings can be reliably translated into clinical practice. A recurring issue is the small sample sizes in most studies, which limits the generalizability of their results. Additionally, the heterogeneity in the study designs, including variability in the population demographics, diabetes subtypes, and methodologies, poses an obstacle to robust comparisons and meta-analyses. These factors emphasize the need for standardization in the research protocols to establish consistent and reproducible results.

A critical limitation is the lack of population stratification. Despite the significant pathophysiological differences between these conditions, many studies fail to differentiate between T1D, T2D, and GD populations. This conflation restricts the development of tailored thresholds and diagnostic criteria for salivary glucose levels, which are likely to vary between diabetes subtypes. Without this differentiation, the interpretation of the findings becomes less precise, potentially obscuring the unique utility of saliva-based monitoring in each context.

Variability in the saliva collection methods also presents a substantial barrier. Differences between stimulated and unstimulated collection techniques and inconsistencies in the pre-collection guidelines introduce variability that complicates the interpretation of the results. External factors such as diet, fasting status, and oral health further influence salivary biomarkers, underscoring the necessity of standardized protocols for collection and analysis. While many studies report moderate to strong correlations between salivary and blood glucose levels, inconsistencies in sensitivity and specificity highlight the need for refined analytical techniques and carefully calibrated cutoff values.

Integrating saliva-based glucose monitoring into clinical practice presents a transformative opportunity to improve diabetes care. This approach offers a painless, non-invasive, and patient-friendly alternative to traditional blood-based tests, potentially enhancing compliance and simplifying glycemic monitoring for populations such as children, pregnant women, and the elderly. However, significant challenges remain. The current methodologies must achieve a higher accuracy and reliability, matching the standards of blood-based diagnostics. Developing cost-effective assays and ensuring their accessibility in diverse healthcare settings is critical, particularly in low-resource areas.

Laboratory workflows must adapt to saliva’s unique composition to successfully implement saliva-based monitoring. Standardized collection protocols, storage methods, and analytical platforms tailored to saliva’s characteristics will be essential. Furthermore, large-scale, multi-center studies are needed to validate the reliability of salivary glucose measurements, refine the diagnostic thresholds, and assess their longitudinal consistency. Such research should also explore the integration of salivary glucose with other biomarkers, such as inflammatory markers and electrolytes, to comprehensively assess a patient’s metabolic state.

The potential for saliva-based methods to replace or supplement traditional blood-based monitoring is nothing short of revolutionary. By reducing the physical and psychological burden of frequent finger pricks, saliva-based methods could significantly improve the quality of life for patients while facilitating more consistent glycemic control. For healthcare systems, these advancements promise better patient compliance, reduced complications, and potentially lower costs associated with diabetes management. However, realizing this vision will require concerted efforts to address the current limitations and establish robust, evidence-based frameworks for clinical application. With continued innovation and rigorous validation, saliva-based monitoring could become a cornerstone of personalized, non-invasive diabetes care.

This scoping review presents limitations inherent to its methodology. The limited availability of research on this topic results in significant gaps, hindering a comprehensive understanding of the relationship between biomarkers. Additionally, the absence of quantitative analyses, such as meta-analyses, prevents the identification of robust statistical correlations, reducing the strength of the conclusions. Another relevant challenge is the heterogeneity of the studies included, as methodological differences, such as variations in the collection and analysis protocols, complicate direct comparisons and make the interpretation of the results more complex.

The viability of salivary glucose as a biomarker depends on the standardization of the analysis methods, as the lack of uniformity in the protocols compromises the reproducibility of studies. Differences in the collection, fasting time, and prior care hinder the comparability of the findings. Standardized guidelines are essential for their clinical and scientific application. Additionally, future research should evaluate its sensitivity and specificity, exploring its viability as an alternative to blood glucose. The validation of more precise methodologies could enable its use as a non-invasive method for glycemic monitoring, benefiting patients with diabetes and other metabolic conditions.

To address the limitations identified in the current research, several advanced strategies can be employed. One promising approach is the utilization of large language models (LLMs), which can enhance the sample size through data augmentation techniques. Moreover, analytical methodologies based on LLMs hold the potential to yield novel insights that the traditional methods may overlook. Implementing blockchain technology could significantly improve data integrity and transparency. Blockchain, an innovative system leveraging distributed storage, peer-to-peer communication, consensus mechanisms, and cryptography, records transactions chronologically to ensure a secure audit trail. Data are structured sequentially and interconnectedly, wherein new entries can be added without the possibility of removal or alteration without a network consensus. This principle of immutability, along with a distributed consensus, safeguards data integrity, drastically mitigating the risks associated with fraud, tampering, or information manipulation [127]. In the context of salivary glucose research, leveraging blockchain and other emerging technologies could address current challenges, thereby enhancing the reliability and clinical applicability of findings. Key solutions include robust data traceability and integrity management, which facilitate the recording and tracking of saliva samples, thereby ensuring data authenticity and immutability—vital for preventing manipulations and bolstering the reliability of research [128,129]. Furthermore, a decentralized platform for global scientific collaboration could emerge through the use of blockchain, creating an accessible decentralized database for researchers worldwide. This would promote open collaboration and reproducibility, allowing for the rapid validation of new discoveries and secure, transparent sharing of the results across different research centers [128,129].

Lastly, future longitudinal clinical studies must tackle a range of critical questions. To support this endeavor, it is essential to formulate a comprehensive study protocol that meticulously outlines the design framework. This protocol should incorporate external variables such as dietary practices, fasting protocols, and oral hygiene, as these factors can significantly influence the results of saliva-based glucose measurements. Additionally, the methodology for saliva collection aimed at glucose analysis needs to adhere to stringent protocols to ensure both the reproducibility and accuracy of the findings. Key aspects to consider include the following:

(A) Type of collection:
  - Stimulated: Saliva is collected by chewing paraffin or sugar-free gum, enhancing flow.
  - Unstimulated: This method relies on the natural saliva flow without external stimulation. Unstimulated saliva is preferred, as it minimizes the effects of dilution and reduces variability in glucose concentrations [17,74].
(B) The metabolic state of the patient: The fasting duration should be standardized to between 8 and 12 h to eliminate fluctuations linked to recent food consumption [15,61].
(C) Pre-collection care: Rigorous oral hygiene protocols are essential. Patients should refrain from brushing their teeth, using mouthwashes, smoking, or consuming any substances that may affect their salivary glucose levels for 30 min before collection. It is also advisable for patients to rinse their mouths with water to eliminate food debris before sample collection [4].
(D) Collection method: The patient should slightly tilt their head forward to promote the accumulation of saliva in the oral cavity before expectorating into a sterile tube [15,74]. To mitigate the degradation of sensitive peptides, samples should be collected into pre-chilled polypropylene tubes maintained on ice [17]. Following their collection, samples should be centrifuged at 3000 RPM for 20 min to yield a clear supernatant for glucose analysis [17,55,58,74].
(E) Storage and processing: Saliva must be stored at specified controlled temperatures. Salivary glucose can be preserved for at least one month at −20 °C; however, its levels begin to decline after two freeze/thaw cycles. For optimal long-term stability, it is recommended to freeze saliva samples in aliquots immediately post-collection at −20 °C [17,58].

Furthermore, exploring the feasibility of integrating multiple biomarkers into a single saliva diagnostic could provide a more holistic view of an individual’s metabolic status.

## 3. Materials and Methods

This scoping review was conducted per the Joanna Briggs Institute (JBI) guidelines for scoping reviews [130]. The PRISMA Extension for Scoping Reviews (PRISMA-ScR) framework provided a structured approach to presenting the results clearly and transparently [131]. The study protocol was registered on the Open Science Framework (OSF), ensuring transparency and replicability. Further details are available at https://doi.org/10.17605/OSF.IO/U5C9W. The following presents the details of the search strategy, the eligibility criteria for study inclusion, the article selection process, and the methodology for extracting data from the selected studies.

### 3.1. The Research Strategy

The research was conducted between May and July 2024, utilizing three databases: PubMed, Scopus, and Web of Science. A comprehensive and customized search strategy was implemented for each database, combining keywords related to diabetes, salivary glucose, and glycemia using the Boolean operators “OR” and “AND”. No restrictions were applied to time or language. A manual search was conducted, examining the reference lists from the included articles and relevant searches via Google (Table 3).

### 3.2. Eligibility Criteria

The eligibility criteria were defined based on a conceptual framework focusing on using salivary glucose as a supportive tool for measuring the glycemic levels in people with diabetes. This review’s central research question was the following: “Is salivary glucose related to glycemic changes in diabetic patients, and can it be used as an auxiliary tool in monitoring this disease?”. Only primary research articles that examined the correlation between salivary glucose levels and blood parameters or evaluated saliva’s utility as a glycemic monitoring tool were included. Studies focusing solely on salivary glucose without linking it to blood glucose or other markers, as well as review articles, editorials, books, and conference abstracts, were excluded.

### 3.3. Study Selection

The article selection process occurred in two stages. Initially, one investigator conducted comprehensive searches across the specified databases, compiling the results on the Rayyan web platform to identify and remove duplicates. Through a blind review process, four reviewers independently screened their titles and abstracts. Articles meeting the inclusion criteria were subjected to a full-text review to assess their suitability based on the predefined eligibility criteria. Excluded articles were documented, and the reasons for exclusion are outlined in Figure 1.

### 3.4. Data Extraction

Data from selected articles were extracted using a standardized form, capturing essential details such as the authorship, publication year, diabetes type, population characteristics, salivary sampling methods, and glucose determination techniques. To improve their visualization and comprehension, the most pertinent data were presented in graphical formats (Figure 2). The extracted findings were critically evaluated and are summarized in Table 2 and Table 3 to highlight the limitations of using and future potential to use salivary glucose as a monitoring strategy. This structured approach ensured a comprehensive synthesis of the current evidence, offering valuable insights into salivary glucose’s application in diabetes management.

## 4. Conclusions

This scoping review illustrates that salivary glucose has significant potential as a non-invasive and accessible biomarker for monitoring glycemic levels in individuals with diabetes. The studies reviewed consistently demonstrate a correlation between salivary glucose levels and blood glucose, affirming the feasibility of utilizing saliva as an alternative fluid for glycemic monitoring. In addition to glucose, other salivary biomarkers, such as proteins, inflammatory markers, AGEs, and electrolytes, provide complementary insights into metabolic health, further enhancing the role of saliva in diabetes care.

Research like this scoping review is crucial to advancing saliva-based monitoring approaches. By synthesizing the existing evidence, identifying methodological gaps, and suggesting future research pathways, this review makes a significant contribution to the scientific community. It highlights saliva’s transformative potential as a strategic biological sample, particularly in diabetes care, and sets the standard for subsequent investigations aimed at validating and refining this innovative approach.

In conclusion, while challenges remain, the potential to use saliva as a reliable sample for glycemic monitoring marks a substantial advancement in diabetes care. With further validation and technological innovation, saliva-based monitoring approaches could become fundamental to personalized, non-invasive, and patient-centered diabetes management, greatly enhancing the quality of life for individuals with this chronic condition.

## Figures and Tables

**Table 1:** Summary of study characteristics and biomarkers.

Author and Year	Type of Diabetes	Number of Diabetic and Non-Diabetic (**♂**/**♀**)	Saliva Sample	Fasting (Yes/No)	Biomarkers
Englander et al., 1963 [23]	Not specified	26 and 26	Stimulated (lemon juice)	Yes	Plasma glucose
Campbell et al., 1965 [24]	Not specified	60 and 60	Spontaneous	No, 2 h after last meal	Plasma glucose, galacturonic acid, glucuronic acid, lactose, and others
Ben-Aryeh et al., 1988 [25]	Not specified	31 (17/14) and 35 (20/15)	Spontaneous and stimulated (citric acid)	No, 1 h after last meal	Plasma glucose, sodium, potassium, protein, and amylase
Darwazeh et al., 1991 [26]	Not specified	41 and 34	Spontaneous	No, 2 h after last meal	Plasma glucose, Hb_A1C_, and candida count
Belazi et al., 1998 [27]	T1D	10 (5/5) and 10 (6/5)	Spontaneous	No, 2 h after last meal	Plasma glucose
Sashikumar et al., 2009 [28]	T2D	100 and 50	Spontaneous and stimulated (citric acid)	No, 2 h after last meal	Plasma glucose, Hb_A1C_, and candida count
Jurysta et al., 2009 [29]	T1D and T2D	84 (36/48) and 38 (16/22)	Spontaneous and stimulated (mastication)	Yes	Plasma glucose
Hegde et al., 2010 [30]	T2D	26 and 21	Spontaneous	Not informed	Plasma glucose, pH, and oxidative stress markers
Vasconcelos et al., 2010 [31]	T2D	40 and 40	Spontaneous	No, 90 min after last meal	Plasma glucose
Panchbhai et al., 2010 [32]	T1D and T2D	40 (16/24) and 80 (25/15 and 22/18)	Spontaneous	No, 2 h after last meal	Plasma glucose
Nagalaxmi et al., 2011 [33]	T1D	50 (28/22) and 50 (28/22)	Spontaneous and draining	Yes	Plasma glucose
Gheena et al., 2011 [34]	T1D	32 and 32	Spontaneous	No, 1 h after last meal	Plasma glucose, cholesterol, albumin, and total protein
Abikshyeet et al., 2012 [35]	T2D	106 (52/54) and 15 (9/6)	Spontaneous	Yes	Plasma glucose and Hb_A1C_
Agrawal et al., 2013 [12]	Not specified	40 and 40	Spontaneous	No, 30 min after last meal	Plasma glucose
Balan et al., 2014 [36]	T2D	30 and 60	Spontaneous	No, 2 h after last meal	Plasma glucose
Kumar et al., 2014 [37]	T2D	60 and 30	Spontaneous	No, 2 h after last meal	Plasma glucose, Hb_A1C_, and salivary candida count
Satish et al., 2014 [22]	T2D	20 and 10	Spontaneous	Yes	Plasma glucose
Shahbaz et al., 2014 [38]	T1D	30 and 30 (30/30)	Spontaneous	Yes	Plasma glucose, total protein, and albumin
Patel et al., 2015 [39]	T1D and T2D	50 and 50	Spontaneous	Not informed	Plasma glucose
Gupta et al., 2015 [40]	T2D	100 (46/54) and 100 (54/46)	Spontaneous	No	Plasma glucose and Hb_A1C_
Arora et al., 2015 [41]	T1D	100 (64/36) and 100 (47/53)	Spontaneous	Yes	Plasma glucose
Lakshmi et al., 2015 [42]	T1D	30 and 30	Spontaneous	Yes	Plasma glucose
Ravindran et al., 2015 [43]	T2D	30 and 30	Spontaneous	Yes	Plasma glucose and Hb_A1C_
Gupta et al., 2015 [44]	T1D and T2D	212 and 38 (106/144)	Spontaneous and aspiration	Yes	Plasma glucose
Indira et al., 2015 [45]	T2D	20 (10/10) and 20 (10/10)	Spontaneous	No, 2 h after last meal	Plasma glucose, salivary amylase, and total protein
Kadashetti et al., 2015 [46]	Not specified	53 (26/27) and 37 (22/15)	Spontaneous	Yes	Plasma glucose
Mussavira et al., 2015 [47]	T2D	53 and 40	Spontaneous	Yes	Plasma glucose, total protein, uric acid, and antioxidant markers
Smriti et al., 2016 [48]	Not specified	120 and 60	Spontaneous	Yes	Plasma glucose
Dhanya et al., 2016 [49]	T2D	100 and 100	Spontaneous	Yes	Plasma glucose
Puttaswamy et al., 2017 [50]	T2D	40 and 60	Spontaneous	Not informed	Plasma glucose
Wang et al., 2017 [51]	T2D	30 (17/13) and 30 (15/15)	Spontaneous	Yes	Plasma glucose
Abd-Elraheem et al., 2017 [52]	T2D	20 and 20 (20/20)	Spontaneous	Yes	Plasma glucose, Hb_A1C_, IgA, and salivary amylase
Shaik et al., 2017 [53]	T2D	70 and 70	Spontaneous	Yes and 2 h postprandial	Plasma glucose
Carramolino-Cuéllar et al., 2017 [54]	T2D	46 and 47	Spontaneous and stimulated (paraffin tablet)	Yes and 2 h postprandial	Plasma glucose
Gupta et al., 2017 [55]	Not specified	80 and 40	Spontaneous	Yes and postprandial	Plasma glucose
Ghafouri et al., 2018 [56]	T2D	50 and 50	Spontaneous	Yes	Plasma glucose
Tiongco et al., 2018 [15]	T2D	97 and 34	Spontaneous	Yes	Plasma glucose
Bhattacharyya et al., 2018 [57]	T2D	97 and 34	Spontaneous	Yes and 2 h postprandial	Plasma glucose
Harish et al., 2019 [58]	T2D	50 and 50	Spontaneous	Yes	Plasma glucose, Hb_A1C_
Fares et al., 2019 [59]	T2D and pre-diabetes	154 and 50	Spontaneous	Not informed	Plasma glucose
Mishra et al., 2019 [60]	T2D	100 and 100	Spontaneous	No, 2 h after last meal	Plasma glucose and candida count
Ephraim et al., 2019 [61]	Not specified	79 and 59	Spontaneous	Yes	Plasma glucose
Ragunathan et al., 2019 [62]	Not specified	40 and 40	Spontaneous	No, 90 min after last meal	Plasma glucose
Hegde et al., 2020 [63]	Not specified	59 and 29 (45/43)	Spontaneous	Not informed	Plasma glucose, IgA, and candida count
Mrag et al., 2020 [64]	T2D	300 and 300	Spontaneous	Yes	Plasma glucose, urea, amylase, total protein, albumin, electrolytes, C-reactive protein (CRP), and immunoglobulin A (IgA)
Kumar et al., 2020 [65]	T1D and T2D	150 and 50	Spontaneous	Not informed	Plasma glucose
Gupta et al., 2020 [66]	Not specified	45 and 45	Spontaneous	Yes and 2 h postprandial	Plasma glucose
Dharmakeerthi et al., 2021 [67]	T2D	120 and 31	Spontaneous	Yes	Plasma glucose
Ganesan et al., 2021 [68]	GD	100 (0/100) and 99 (0/99)	Spontaneous and stimulated (citric acid)	Yes and 2 h postprandial	Plasma glucose
Egboh et al., 2022 [69]	T2D	45 (23/22) and 40 (20/20)	Spontaneous	Yes	Plasma glucose, sodium, potassium, bicarbonate, and chlorine
Cui et al., 2022a [17]	T1D and T2D	40 and 40	Spontaneous and stimulated (citric acid)	No, 30 min after last meal	Plasma glucose
Cui et al., 2022b [4]	Not specified	40 and 40	Spontaneous	No, 30 min after last meal	Plasma glucose
Ganesan et al., 2022 [70]	T1D	79 (38/41) and 100 (58/42)	Spontaneous and stimulated (citric acid)	Yes and 2 h postprandial	Plasma glucose
Cheprasova et al., 2022 [71]	T1D	40 (20/20) and 40 (20/20)	Spontaneous	Not informed	Plasma glucose, salivary protein, salivary cholesterol and triglycerides, and chaperone-like activity
Choudhry et al., 2022 [72]	T2D	100 (67/33) and 100 (65/35)	Spontaneous	Yes	Plasma glucose
Pandey et al., 2023 [73]	T1D, T2D and GD	200 (105/95) and 200 (102/98)	Spontaneous	Not informed	Plasma glucose
Shettigar et al., 2024 [74]	T2D	83 (31/52) and 83 (36/47)	Spontaneous	Not informed	Plasma glucose

Abbreviations: T1D: type 1 diabetes; T2D: type 2 diabetes; CFU: candida colony-forming units.

**Table 2:** Comparison of fasting serum and salivary glucose levels.

Author and Year	Type of Diabetes	Fasting Serum Glucose (mg/dL) (DM/Control)		Fasting Salivary Glucose (mg/dL) (DM/Control)	
Englander et al., 1963 [23]	Not specified	142	92	1.61	0.78
Campbell et al., 1965 [24]	Not specified	-	-	-	-
Ben-Aryeh et al., 1988 [25]	Not specified				
Darwazeh et al., 1991 [26]	Not specified	-	-	-	-
Belazi et al., 1998 [27]	T1D	-	-	-	-
Sashikumar et al., 2009 [28]	T2D	-	-	-	-
Jurysta et al., 2009 [29]	T1D and T2D	-	-	Stimulated: ♂ 3.67/♀ 3.15 Not Stimulated: ♂ 3.64/♀ 3.15	Stimulated: ♂ 0.53/♀ 0.62 Not Stimulated: ♂ 1.42/♀ 1.45
Hegde et al., 2010 [30]	T2D	144.31	99.71	10.46	7.41
Vasconcelos et al., 2010 [31]	T2D	-	-	-	-
Panchbhai et al., 2010 [32]	T1D and T2D	-	-	-	-
Nagalaxmi et al., 2011 [33]	T1D	306.1	86.56	30.54	9.174
Gheena et al., 2011 [34]	T1D	-	-	-	-
Abikshyeet et al., 2012 [35]	T2D	154.70	86.82	4.22	1.23
Agrawal et al., 2013 [12]	Not specified	171.31	92.11	10.93	6.08
Balan et al., 2014 [36]	T2D	-	-	-	-
Kumar et al., 2014 [37]	T2D	-	-	-	-
Satish et al., 2014 [22]	T2D	205.2	90.5	12.11	4.32
Shahbaz et al., 2014 [38]	T1D	213.8	82.96	2.1	0.813
Patel et al., 2015 [39]	T1D and T2D	167.06	78.94	13.96	4.61
Gupta et al., 2015 [40]	T2D	-	-	-	-
Arora et al., 2015 [41]	T1D	204.44	82.02	20.14	7.65
Lakshmi et al., 2015 [42]	T1D	-	-	8.56	5.06
Ravindran et al., 2015 [43]	T2D	230.067	92.50	6.567	1.867
Gupta et al., 2015 [44]	T1D and T2D	T1D = 217.62 T2D = 174.24 Both = 183.86	84.18	T1D = 10.21 T2D = 9.92 Both = 9.98	6.8
Indira et al., 2015 [45]	T2D	-	-	-	-
Kadashetti et al., 2015 [46]	Not specified	-	-	Group III (<130 mg/dL): 5.78 Group II (130–200 mg/dL): 9.81 Group I (>200 mg/dL): 15.5	-
Mussavira et al., 2015 [47]	T2D	Controlled: 109 Uncontrolled: 211.85 All: 161.07	86.30	Controlled: 8.34 Uncontrolled: 3.41 All: 5.83	2.07
Smriti et al., 2016 [48]	Not specified	-	-	Not Medicated = 11.68 Medicated = 9.68	6.5
Dhanya et al., 2016 [49]	T2D	136.30	97.78	8.47	1.20
Puttaswamy et al., 2017 [50]	T2D	-	-	-	-
Wang et al., 2017 [51]	T2D	134.41	100.15	Parotid: 3.24 Mix: 0.57	Parotid: 1.39 Mix: 0.62
Abd-Elraheem et al., 2017 [52]	T2D	-	-	10.9	4.88
Shaik et al., 2017 [53]	T2D	201.471	101.614	7.634	5.469
Carramolino-Cuéllar et al., 2017 [54]	T2D	-	-	Not Stimulated: 5.57 Stimulated: 4.31	Not Stimulated: 3.73 Stimulated: 3.46
Gupta et al., 2017 [55]	Not specified	Controlled: 121.53 Uncontrolled: 283.23	78.39	Controlled: 4.86 Uncontrolled: 11.33	0.78
Ghafouri et al., 2018 [56]	T2D	161.00	74.75	12.80	6.5
Tiongco et al., 2018 [15]	T2D	preDM = 115.8 T2D = 189.1	93.7	preDM = 10.5 T2D = 16.3	5.3
Bhattacharyya et al., 2018 [57]	T2D	Controlled 96.62 Uncontrolled 170.76	92.51	Controlled: 9.14 Uncontrolled: 15.21	7.18
Harish et al., 2019 [58]	T2D	Controlled 103 Uncontrolled 162	91.88	Controlled: 4.75 Uncontrolled: 6.07	4.27
Fares et al., 2019 [59]	T2D and pre-diabetes	DM = 226.89 PreDM = 111.31	86.45	DM = 59.32 PreDM = 42.68	23.40
Mishra et al., 2019 [60]	T2D	-	-	-	-
Ephraim et al., 2019 [61]	Not specified	285.22	88.28	16.58	5.76
Ragunathan et al., 2019 [62]	Not specified	-	-	-	-
Hegde et al., 2020 [63]	Not specified	-	-	-	-
Mrag et al., 2020 [64]	T2D	180.18	78.38	7.21	3.6
Kumar et al., 2020 [65]	T1D and T2D	-	-	-	-
Gupta et al., 2020 [66]	Not specified	194.53	74.71	1	1.2
Dharmakeerthi et al., 2021 [67]	T2D	163.03	95.24	1.38	0.36
Ganesan et al., 2021 [68]	GD	-	-	-	-
Egboh et al., 2022 [69]	T2D	180.4		19.63	11.15
Cui et al., 2022a [17]	T1D and T2D	-	-	-	-
Cui et al., 2022b [4]	Not specified	-	-	-	-
Ganesan et al., 2022 [70]	T1D	142.11	87.98	6.04	1.46
Cheprasova et al., 2022 [71]	T1D	♂ 183.4 ♀ 169.65	♂ 95.04 ♀ 92.7	♂ 11.84 ♀ 12.22	♂ 3.07 ♀ 3.07
Choudhry et al., 2022 [72]	T2D	183.36	79.6	4.37	0.92
Pandey et al., 2023 [73]	T1D, T2D, and GD	228.94	96.13	13.93	5.76
Shettigar et al., 2024 [74]	T2D	-	-	-	-

Abbreviations: DM: diabetes mellitus; T1D: type 1 diabetes; T2D: type 2 diabetes.

**Table 3:** Search strategies for PubMed, Scopus, and Web of Science articles.

Database	Queries
PubMed	(“Diabetes Mellitus”[MeSH Terms] OR “diabetes mellitus, type 1”[MeSH Terms] OR “diabetes mellitus, type 2”[MeSH Terms] OR “diabetes, gestational”[MeSH Terms] OR “Diabetes Mellitus”[Title/Abstract] OR “DM”[Title/Abstract] OR “Type 1 diabetes”[Title/Abstract] OR “T1D”[Title/Abstract] OR “Type 2 diabetes”[Title/Abstract] OR “T2D”[Title/Abstract] OR “Gestational diabetes”[Title/Abstract]) AND (“serum glucose”[Title/Abstract] OR “blood glucose”[Title/Abstract] OR “plasma glucose”[Title/Abstract]) AND (“saliva”[MeSH Terms] OR “saliva”[Title/Abstract] OR (“salivary glucose”[Title/Abstract] OR “salivary”[Title/Abstract]))
Scopus	(TITLE-ABS-KEY (“diabetes mellitus” OR “DM” OR “type 1 diabetes” OR “T1D” OR “type 2 diabetes” OR “T2D” OR “gestational diabetes”)) AND (TITLE-ABS-KEY (“serum glucose” OR “blood glucose” OR “plasma glucose”)) AND (TITLE-ABS-KEY (“saliva” OR “salivary glucose” OR “salivary”))
Web of Science	(TS=(“diabetes mellitus”)) OR TS=(DM)) OR TS=(“Type 1 diabetes”)) OR TS=(“Type 2 diabetes”)) OR TS=(“Gestational diabetes”)) OR TS=(“T1D”)) OR TS=(“T2D”) AND (TS=(“serum glucose”)) OR TS=(“blood glucose”)) OR TS=(“plasma glucose”) AND (TS=(“saliva”)) OR TS=(“salivary glucose”)) OR TS=(“salivary”)

## Data Availability

The authors confirm that the data supporting the findings of this study are available within the article.

## Supplementary Materials

The following supporting information can be downloaded at https://www.mdpi.com/article/10.3390/biomedicines13030713/s1. Table S1: Data extraction from included studies.

## Abbreviations

DM	Diabetes mellitus
T1D	Type 1 diabetes
T2D	Type 2 diabetes
ADA	American Diabetes Association
GD	Gestational diabetes
Hb_A1c_	Glycated hemoglobin
CRP	C-reactive protein
IL-6	Interleukin-6
IgA	Immunoglobulin A
TNF-α	Tumor necrosis factor-alpha
OGTT	Oral Glucose Tolerance Test
AGEs	Advanced glycation end products
AUC	Area under the curve
LLMs	Large language models
CFU	Candida colony-forming units
LADA	Latent Autoimmune Diabetes in Adults
UWS	Unstimulated whole saliva
SWS	Stimulated whole saliva
USS	Unstimulated sublingual saliva
SSS	Stimulated sublingual saliva
UPS	Unstimulated parotid saliva
SPS	Stimulated parotid saliva
